# Serum Interleukin-6 and interleukin-8 are early biomarkers of acute kidney injury and predict prolonged mechanical ventilation in children undergoing cardiac surgery: a case-control study

**DOI:** 10.1186/cc7940

**Published:** 2009-07-01

**Authors:** Kathleen D Liu, Christopher Altmann, Gerard Smits, Catherine D Krawczeski, Charles L Edelstein, Prasad Devarajan, Sarah Faubel

**Affiliations:** 1 Divisions of Nephrology and Critical Care Medicine, Departments of Medicine and Anesthesia, University of California, San Francisco, San Francisco, CA, USA; 2 Division of Renal Diseases and Hypertension, University of Colorado Health Sciences Center, University of Colorado Denver, Aurora, CO, USA; 3 Section of Cardiology, Cincinnati Children's Hospital Medical Center, University of Cincinnati School of Medicine, Cincinnati, OH, USA; 4 Section of Nephrology and Hypertension, Cincinnati Children's Hospital Medical Center, University of Cincinnati School of Medicine, Cincinnati, OH, USA

## Abstract

**Introduction:**

Acute kidney injury (AKI) is associated with high mortality rates. New biomarkers that can identify subjects with early AKI (before the increase in serum creatinine) are needed to facilitate appropriate treatment. The purpose of this study was to test the role of serum cytokines as biomarkers for AKI and prolonged mechanical ventilation.

**Methods:**

This was a case-control study of children undergoing cardiac surgery. AKI was defined as a 50% increase in serum creatinine from baseline within 3 days. Levels of serum interleukin (IL)-1β, IL-5, IL-6, IL-8, IL-10, IL-17, interferon (IFN)-γ, tumor necrosis factor-α (TNF-α), granulocyte colony-stimulating factor (G-CSF), and granulocyte-macrophage colony-stimulating factor (GM-CSF) were measured using a bead-based multiplex cytokine kit in conjunction with flow-based protein detection and the Luminex LabMAP multiplex system in 18 cases and 21 controls. Levels of IL-6 and IL-8 were confirmed with single-analyte ELISA; IL-18 was also measured with single-analyte ELISA.

**Results:**

IL-6 levels at 2 and 12 hours after cardiopulmonary bypass (CPB) and IL-8 levels at 2, 12 and 24 hours were associated with the development of AKI using the Wilcoxon rank-sum test and after adjustment for age, gender, race, and prior cardiac surgery in multivariate logistic regression analysis. In patients with AKI, IL-6 levels at 2 hours had excellent predictive value for prolonged mechanical ventilation (defined as mechanical ventilation for more than 24 hours postoperatively) by receiver operator curve (ROC) analysis, with an area under the ROC curve of 0.95. IL-8 levels at 2 hours had excellent predictive value for prolonged mechanical ventilation in all patients. Serum IL-18 levels were not different between those with and without AKI.

**Conclusions:**

Serum IL-6 and IL-8 values identify AKI early in patients undergoing CPB surgery. Furthermore, among patients with AKI, high IL-6 levels are associated with prolonged mechanical ventilation, suggesting that circulating cytokines in patients with AKI may have deleterious effects on other organs, including the lungs.

## Introduction

Acute kidney injury (AKI) in hospitalized patients is associated with unacceptably high mortality rates (in the range of 30% to 50% in most recent series for dialysis-requiring AKI) [1,2]. In addition, the costs associated with AKI are high, as AKI translates into longer lengths of stay as well as a frequent need for invasive procedures (*e.g*., line placement and dialysis).

At present, therapies for AKI are limited to supportive care, such as dialysis. A number of major impediments exist to developing therapies for AKI. First, biomarkers that diagnose AKI before an increase in serum creatinine are needed (reviewed in [3,4]). Because serum creatinine is a marker of glomerular filtration rate and therefore of established AKI, substantial kidney injury may have occurred by the time serum creatinine increases. Second, the pathogenesis of AKI in humans is complex and involves the endothelial and epithelial cell compartments, as well as inflammatory cells. Finally, AKI may have a detrimental impact on other organs, particularly the lung [5-7]. Predicting distant organ injury is critical to developing better therapies for AKI, because other end-organ injury may be a major mechanism for morbidity and mortality related to AKI.

AKI is associated with inflammation. In patients with established AKI, serum interleukin (IL)-6, IL-8, IL-1β, IL-10 and tumor necrosis factor-α (TNF-α), were increased [8]. In an animal model of AKI, we demonstrated that inflammatory cytokines increase *early *after AKI as serum interleukin-6 (IL-6) and keratinocyte-derived cytokine (KC, the murine analogue of interleukin-8) were increased by 2 hours after AKI [9]. Whether these and other cytokines might be early biomarkers of AKI in patients, and whether these biomarkers would predict other adverse outcomes in patients with AKI are unknown. To test whether serum cytokines might be early biomarkers of AKI, we examined serum IL-1β, IL-5, IL-6, IL-8, IL-10, IL-17, IL-18, interferon (IFN)-γ, TNF-α, granulocyte colony-stimulating factor (G-CSF), and granulocyte-macrophage colony-stimulating factor (GM-CSF) in pediatric patients with and without AKI, 2, 12, and 24 hours after cardiopulmonary bypass (CPB). Based on our animal data, we hypothesized that IL-6 and IL-8 would be early biomarkers of acute kidney injury.

In animals, we and others demonstrated that AKI causes lung injury, characterized by neutrophil infiltration and increased capillary permeability [6,9-14]. Furthermore, we recently demonstrated that IL-6 mediates lung injury after both ischemic AKI and bilateral nephrectomy, and that this effect may be dependent on KC (the murine analogue of IL-8) [15]. Therefore, we also hypothesized that early biomarkers of AKI (*e.g*., IL-6 and IL-8) would predict the need for prolonged mechanical ventilation in this study.

## Materials and methods

### Study subjects

All children undergoing correction of congenital heart disease at Cincinnati Children's Hospital between January 2004 and November 2004 were eligible. Exclusion criteria included pre-existing renal insufficiency, diabetes mellitus, peripheral vascular disease, and use of nephrotoxic drugs before or during the study period. Written informed consent was obtained from the legal guardian of each child; the study was approved by the Cincinnati Children's Hospital Institutional Review Board. This study population was previously described in detail [16,17]. As part of standard management, children were treated with a one-time dose of 30 mg/kg methylprednisolone on the CPB pump, with a maximum dose of 500 mg. All of the children received modified ultrafiltration per protocol at the end of surgery. All study subjects received intravenous fluids per a standard protocol (80% of maintenance fluids on postoperative day 1 and 100% of maintenance fluids on subsequent postoperative days). None of the patients had oliguria. Weaning from mechanical ventilation and extubation occurred per protocol.

### Study procedures

Serum creatinine was measured at baseline and at least twice a day postoperatively and at least daily after postoperative day 3. Blood samples were collected at baseline and at 2, 12, and 24 hours after the initiation of CPB, and then once daily for 5 days. When the CPB time was less than 2 hours, the first postoperative serum samples were obtained at the end of CPB, and this sample was considered the 2-hour sample. The primary outcome variable was development of AKI, defined as a 50% or greater increase in serum creatinine from baseline within 3 days. Other variables obtained included age, sex, ethnic origin, CPB time, previous heart surgery, urine output, and duration of mechanical ventilation.

### Statistical analysis

Baseline characteristics and cytokine levels of subjects who did and did not develop acute kidney injury were compared. Categoric variables were expressed as proportions and compared by using the χ^2 ^test. Continuous variables were expressed as mean ± standard deviation or median with interquartile range and were compared by using Student's *t *test or the Wilcoxon rank-sum test, where appropriate.

We next examined the association between biomarker measurements (predictor) and acute kidney injury or prolonged mechanical ventilation (outcomes), by using multivariable logistic regression to adjust for other covariates. Biomarker levels were log transformed because these were not normally distributed. We adjusted for age, sex, race, and operative characteristics. Model discrimination was assessed using ROC curves [18]. Model fit (calibration) was assessed using the Hosmer-Lemeshow goodness-of-fit test, which compares model performance (observed *vs*. expected) across deciles of risk. A nonsignificant value for the Hosmer-Lemeshow χ^2 ^suggests an absence of biased fit. Data analysis was conducted by using Stata 10 (StataCorp, College Station, TX, USA). A *P *value of less than 0.20 was considered potentially significant for interaction. In other cases, two-tailed *P *values less than 0.05 were considered significant.

### Flow cytometry and enzyme-linked immunoassay (ELISA) determination for serum cytokines

Serum IL-1β, IL-5, IL-6, IL-8, IL-10, IL-17, IFN-γ, TNF-α, G-CSF, and GM-CSF were measured in duplicate using a bead-based multiplex cytokine kit (Bio-Rad, Hercules, CA, USA) in conjunction with flow-based protein detection and the Luminex LabMAP multiplex system (Luminex, Austin, TX, USA) according to the manufacturers' directions. The detection limit for each cytokine was 1.95 pg/ml. To confirm results obtained with the multiplex cytokine array, serum IL-6 and IL-8 were measured in duplicate by the appropriate single ELISA (R&D Systems, Minneapolis, MN, USA). The lower limit of detection for IL-6 is less than 0.7 pg/ml, and the detection limit for IL-8 is 1.5 to 7.5 pg/ml. Serum IL-18 was measured in duplicate by single ELISA (Medical and Biologic Laboratories, Nagoya, Japan); the detection limit for IL-18 is 25 pg/ml.

## Results

### Patient characteristics

This was a nested case-control study of a cohort of children undergoing CPB for correction of congenital heart disease. The cohort of patients was previously described and consists of patients with clear ischemic acute kidney injury due to CPB [16,17]. In brief, 100 consecutive children undergoing CPB surgery were considered for study; 29 were excluded for nephrotoxin use. Acute kidney injury (AKI) was defined by a 50% or greater increase in serum creatinine within a 3-day postoperative period. Of the 71 eligible study subjects, AKI developed in 20 patients. Eighteen of the AKI subjects had sufficient serum remaining for analysis of cytokines; 21 controls were selected from the patients without AKI.

No differences were found between subjects in whom AKI developed and those in whom it did not with regard to age, sex, ethnicity, or baseline creatinine (Table 1). AKI was associated with longer CPB times (*P *= 0.0005). A strong association was noted between AKI and the need for prolonged mechanical ventilation, defined as ventilation for more than 24 postoperative hours (*P *= 0.009). Cardiac surgical procedures in children with and without AKI are detailed in Additional data file # 1.

**Table 1:** Baseline characteristics of patients with and without acute kidney injury

	No acute kidney injury	Acute kidney injury	*P *value
Number	21	18	
Age (years)*	2.1 ± 2	3 ± 5.2	0.46
Male (%)	62%	50%	0.46
White (%)	86%	83%	0.84
Redo surgery	24%	39%	0.31
CPB time (minutes)*	87 ± 44	136 ± 63	0.007
Baseline Cr (mg/dl)*	0.4 ± 0.08	0.4 ± 0.17	0.92
Peak Cr (mg/dl)*	0.4 ± 0.11	0.9 ± 0.58	0.0007
CVP (mm Hg)*‡	10.6 ± 2.6	10.6 ± 3.4	0.99
Mechanical ventilation at 24 h (%)	33%	78%	0.006
Hospital length of stay (days)†	4 [3,6]	10.5 [8,30]	<0.0001
Death (%)	0	11%	0.12

*Mean ± SD

† Median [25, 75% interquartile range].

‡ Measured 2 hours after CPB.

### Serum cytokine levels and AKI

Serum IL-1β, IL-5, IL-6, IL-8, IL-10, IL-17, IFN-γ, TNF-α, G-CSF, and GM-CSF were measured at baseline (before CPB) and at 2, 12, and 24 hours after CPB with a multiplex protein-detection method. Compared with AKI-free controls, patients with AKI had significantly increased serum IL-6 and IL-8 levels. No significant differences were observed for IL-1β, IL-5, IL-10, IL-17, IFN-γ, TNF-α, G-CSF, or GM-CSF at any time point (data not shown). Serum IL-18, as measured with ELISA, was also not different between patients with *versus *those without AKI.

**Figure 1:**
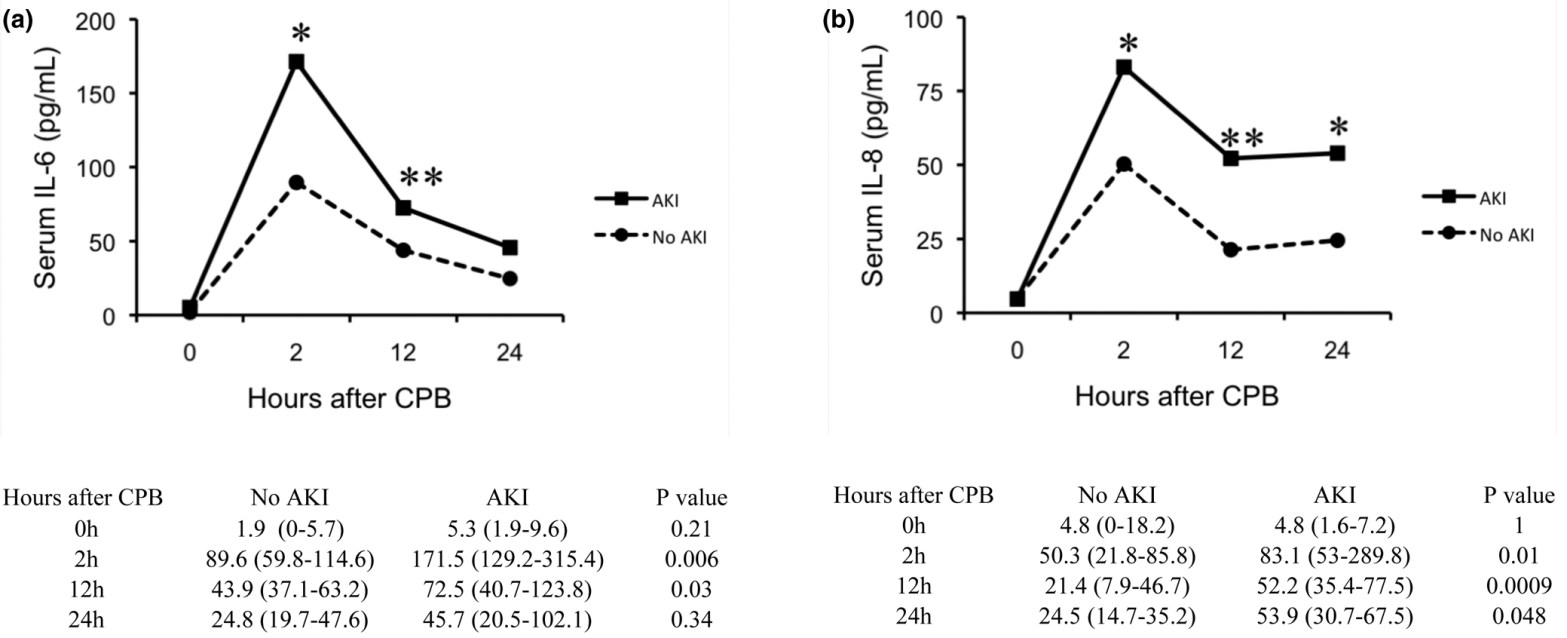
Serum IL-6 and IL-8 are increased in patients with acute kidney injury (AKI) following cardiopulmonary bypass (CPB). **(a) **Serum IL-6 was determined at 0, 2, 12, and 24 hours after cardiopulmonary bypass, and median levels were significantly increased 2 and 12 hours after CPB in patients with AKI *versus *patients without AKI. *P < 0.01; **P < 0.05. **(b) **Serum IL-8 was determined at 0, 2, 12, and 24 hours after cardiopulmonary bypass, and median levels were significantly increased at 2, 12, and 24 hours in patients with AKI *versus *patients without AKI. *P < 0.05; **P < 0.001.

As shown in Figure 1, levels of IL-6 and IL-8 by single-analyte ELISA were not different at the time of CPB between children in whom AKI developed and those in whom it did not. IL-6 and IL-8 levels peaked in both groups at 2 hours after CPB. IL-6 levels were significantly higher in children with AKI at 2 and 12 hours, compared with those without AKI. IL-8 levels were significantly higher in children with AKI at 2, 12, and 24 hours after CPB.

In bivariate analysis, IL-6 and IL-8 levels at 2 and 12 hours were independently associated with the development of acute kidney injury (Table 2). After adjustment for age, sex, race, and whether the patient had previous surgery, IL-6 levels at 2 hours and IL-8 levels at 2 and 12 hours remained predictive for AKI. Because prolonged CPB time is a known risk factor for AKI, and because we hypothesized that high inflammatory cytokine levels are the result of AKI, we specifically chose not to adjust for CPB time in our multivariable model. Alternatively, one of the pathogenetic mechanisms for AKI after CPB is through inflammatory processes mediated by IL-6 and IL-8; thus, cytokine levels and CPB time would not be expected to have independent predictive value in a model for AKI. Similarly, because IL-6 and IL-8 likely represent a common inflammatory pathway, we did not adjust for both cytokines in the same predictive model. Last, we examined the performance of various cut points in cytokine levels for the diagnosis of AKI (Table 3).

**Table 2:** Association between serum IL-6 and IL-8 levels and acute kidney injury

Cytokine	Odds ratio	95% CI	*P *value	Odds ratio*	95% CI	*P *value
IL-6 at 2 hours	3.56	1.28–9.90	0.02	3.47	1.21–9.97	0.02
IL-6 at 12 hours	3.17	1.04–9.66	0.04	3.29	0.86–12.64	0.08
IL-6 at 24 hours	1.45	0.67–3.15	0.34	1.48	0.57–3.80	0.42
IL-8 at 2 hours	2.87	1.25–6.55	0.01	4.98	1.39–17.86	0.01
IL-8 at 12 hours	4.37	1.40–13.64	0.01	9.74	1.75–54.20	0.009
IL-8 at 24 hours	2.44	0.85–6.97	0.10	4.98	0.85–29.15	0.08

Logistic regression was used to determine the association between IL-6 and IL-8 levels at various times after cardiopulmonary bypass and the diagnosis of acute kidney injury. Because biomarker levels were abnormally distributed, they were log transformed, and odds ratios represent the increase in risk per log increase in biomarker level.

*Adjusted for age, sex, race, and previous surgery

**Table 3:** Performance of serum IL-6 and IL-8 for the diagnosis of acute kidney injury at various times after cardiopulmonary bypass

	Sensitivity (%)	Specificity (%)	Positive predictive value (%)*	Negative predictive value (%)*	Area under the ROC curve
IL-6 at 2 hours					0.76
≥175 pg/ml	44	85	63	73	
≥125 pg/ml	78	80	69	87	
≥75 pg/ml	83	25	39	73	
					
IL-6 at 12 hours					0.71
≥175 pg/ml	17	100	100	68	
≥125 pg/ml	22	100	100	70	
≥75 pg/ml	50	89	72	76	

IL-8 at 2 hours					0.74
≥100 pg/ml	44	80	56	72	
≥70 pg/ml	67	65	52	78	
≥40 pg/ml	83	45	46	83	
					
IL-8 at 12 hours					0.82
≥100 pg/ml	18	100	100	68	
≥70 pg/ml	35	100	100	73	
≥40 pg/ml	71	74	60	82	

*Because positive and negative predictive values will vary based on AKI prevalence, we assumed a prevalence of 36%, as in [17].

### Serum cytokine levels and mechanical ventilation

**Figure 2:**
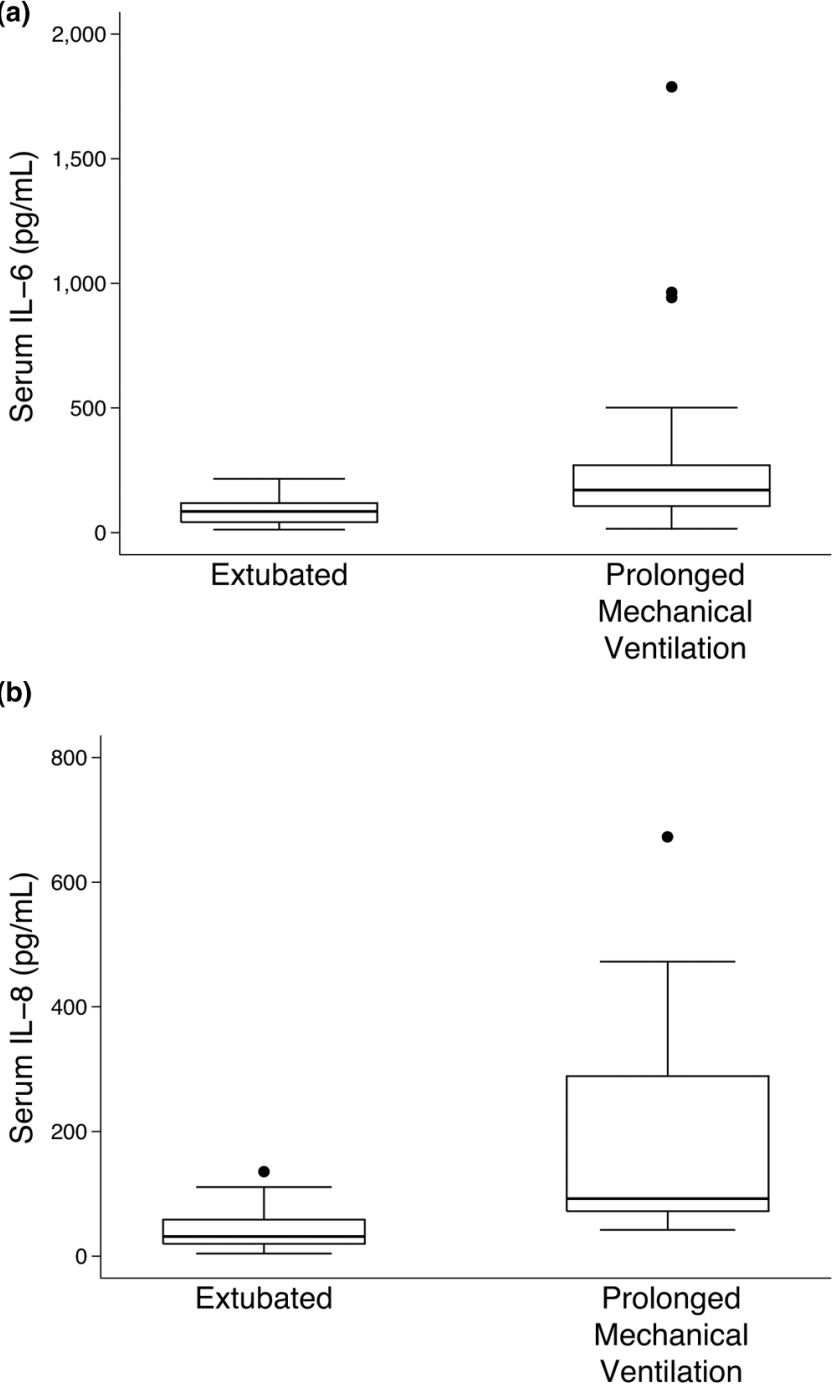
Serum IL-6 and IL-8 are increased in patients who required prolonged mechanical ventilation after cardiopulmonary bypass (CPB). **(a) **Serum IL-6 levels at 2 hours after CPB were significantly increased in patients who required mechanical ventilation at 24 hours after CPB, compared with those who were extubated; *P *= 0.005. (The horizontal line represents the median; box encompasses the 25th through 75th percentiles; and whiskers encompass the 10th through 90th percentiles). **(b) **Serum IL-8 levels at 2 hours after CPB were significantly increased in patients who required mechanical ventilation at 24 hours after CPB, compared with those who were extubated; *P *= 0.0001.

We next compared cytokine levels between children who required prolonged mechanical ventilation, defined as ventilation for more than 24 postoperative hours, and those who did not. Median IL-6 levels at 2 hours after CPB were significantly higher in children who required prolonged mechanical ventilation, compared with those who did not (171 pg/ml [25% to 75% IQR 106.2, 270.3] *vs*. 85.3 pg/ml [41.8, 118.2], *P *= 0.005; Figure 2). Similarly, IL-8 levels at 2 hours after CPB were significantly higher in children who required prolonged mechanical ventilation (92.2 pg/ml [72.1, 288.7] *vs*. 31.3 pg/ml [19.7, 58.6], *P *= 0.0001). IL-6 and IL-8 levels also differed significantly between the two groups at 12 and 24 hours (data not shown).

**Figure 3:**
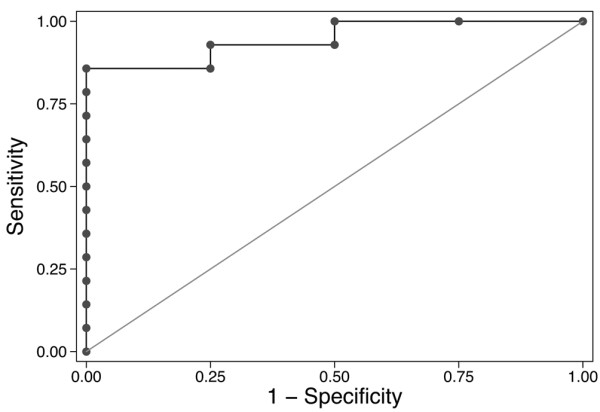
Receiver-operator characteristic (ROC) curve for the ability of serum IL-6 to predict prolonged mechanical ventilation in patients with acute kidney injury (AKI) after cardiopulmonary bypass (CPB). Prolonged mechanical ventilation was defined as more than 24 hours of ventilation. Interleukin-6 levels were log transformed because they were abnormally distributed. The area under the ROC curve is 0.95, with a Hosmer-Lemeshow goodness-of-fit *P *value of 0.85, demonstrating that increased IL-6 at 2 hours is an excellent predictor of prolonged mechanical ventilation in patients with AKI after CPB.

**Figure 4:**
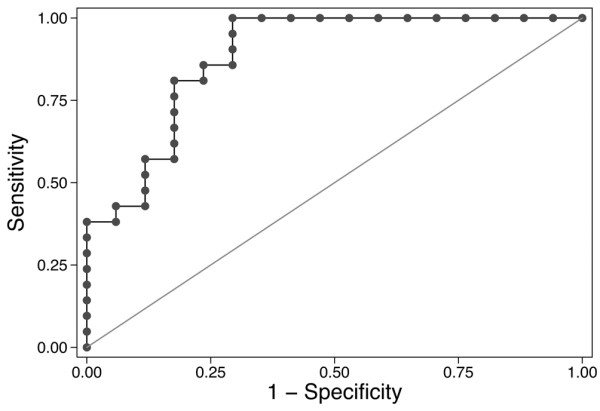
Receiver-operator characteristic (ROC) curve for the ability of IL-8 to predict prolonged mechanical ventilation after cardiopulmonary bypass (CPB). Prolonged mechanical ventilation was defined as more than 24 hours of ventilation. Interleukin-8 levels were log-transformed because they were abnormally distributed. The area under the ROC curve is 0.89, with a Hosmer-Lemeshow goodness-of-fit *P *value of 0.75, demonstrating that increased serum IL-8 at 2 hours is an excellent predictor of prolonged mechanical ventilation in patients after CPB.

When we analyzed the association between cytokine levels and the requirement for prolonged mechanical ventilation, an interaction between IL-6 levels and acute kidney injury was detected (*P *= 0.06). An interaction was not detected between IL-8 levels and acute kidney injury (*P *= 0.83). We therefore stratified the analysis of IL-6 levels by the presence or absence of AKI. IL-6 levels were associated with prolonged mechanical ventilation only in study subjects with acute kidney injury (*P *= 0.008 *vs*. *P *= 0.9 in those without AKI). Indeed, IL-6 levels at 2 hours had excellent predictive value for prolonged mechanical ventilation in patients with AKI, with an area under the ROC curve of 0.95 (Figure 3). IL-8 levels at 2 hours had excellent predictive value for prolonged mechanical ventilation in all patients, with an area under the ROC curve of 0.89 (Figure 4).

## Discussion

In the present study, we have demonstrated that, in children undergoing CPB, AKI is characterized by high levels of serum IL-6 and IL-8. IL-6 and IL-8 levels at 2 and 12 hours after CPB were predictive for subsequent AKI. Furthermore, among children with AKI, early increases in serum IL-6 are predictive of prolonged mechanical ventilation.

We previously demonstrated in animal models that early AKI is characterized by high serum IL-6 and IL-8 [9]. Our study is the first in patients to suggest that early AKI (*i.e*., within 2 hours of the original insult) is a proinflammatory state. Whereas other studies have demonstrated that increased serum IL-6 predicts subsequent AKI [19,20], these studies were conducted in critically ill patients with severe sepsis and acute lung injury. In those studies, the timing of the underlying AKI insult was less clear because of the underlying severity of illness of study subjects. IL-6 was elevated between 1 and 7 days before the detection of AKI, so the timing of the IL-6 elevation relative to AKI was also less clear. Thus, AKI may have contributed to high levels of IL-6, or may have been the result of the patient's proinflammatory state.

In our study, patients were children undergoing CPB, in which the major insult is the surgery and bypass itself. Thus, the timing of the insult is clear. Based on our animal studies, we hypothesized that levels of proinflammatory cytokines would increase early after the ischemic insult (*e.g*., CPB). Indeed, levels of IL-6 and IL-8 were elevated 2 hours after CPB in patients with AKI, well before a detectable increase in creatinine. These results are similar to those observed with other urine and plasma biomarkers of AKI that have been measured in this cohort, including urinary IL-18, serum/urinary neutrophil-gelatinase-associated lipocalin (NGAL), urinary kidney injury molecule-1 (KIM-1), and urinary liver fatty acid-binding protein (L-FABP) [16,17,21,22].

Although CPB is associated with an increase in proinflammatory cytokines [23], data are accumulating that AKI may affect both the production and clearance of cytokines. For example, in animal models of ischemic AKI, increased renal production of both IL-6 and KC (the murine analogue of IL-8) have been documented [9,24,25]. Increased serum cytokines also are detected after bilateral nephrectomy [9], a model of renal failure in which both kidneys are removed, and therefore, the kidney cannot be a source of increased serum cytokines in this model. Thus, extrarenal production of cytokines or impaired clearance of cytokines may also occur in acute renal failure and contribute to elevated serum levels. In this regard, pharmacokinetic studies in animals demonstrated that the kidney plays a key role in the clearance of cytokines [26-28]. In patients, a negative correlation has been demonstrated between serum IL-6 levels and glomerular filtration rate [29], further implicating the kidney in cytokine clearance. Available evidence suggests that cytokines are cleared by the kidney predominantly through filtration, resorption, and metabolism by the proximal tubule [30], although filtration and excretion of the intact protein can occur [9]. In our study, concomitant AKI resulted in a greater than threefold increase in serum IL-6 and IL-8 2 hours after CPB *versus *CPB alone. Thus, although serum cytokines increase after CPB itself, the increase is much greater in the presence of AKI and may be due to decreased clearance or increased production or both.

Mechanical ventilation is a consistent, independent predictor of mortality in patients with AKI [31-35], and a recent study demonstrated that patients with AKI required mechanical ventilation for more days than did patients with similar severity of illness who did not have AKI [36]. The reasons for the prolonged duration of mechanical ventilation in patients with AKI is unknown. In mice, IL-6 signalling effects are increased in the lung after AKI [37], and our recently published data demonstrate that IL-6 mediates lung injury after AKI, as IL-6-deficient and IL-6 antibody-treated mice had reduced lung inflammation, capillary leak, and serum and lung KC after AKI [15]. Although the role of IL-6 in other forms of lung injury has not been examined, a pathogenic role of IL-6 in ventilator-associated lung injury has been hypothesized [38]; patients receiving lung-protective ventilation (6 ml/kg tidal volume) had lower serum IL-6 levels, which predicted reduced mortality and more ventilator-free days *versus *patients receiving standard ventilation (12 ml/kg tidal volume) [39]. In the present study, we demonstrate that in patients with AKI, increased serum IL-6 2 hours after CPB was predictive for prolonged mechanical ventilation. Recognizing that we are unable to prove causality in this context, we hypothesize that AKI directly contributes to prolonged mechanical ventilation, perhaps through higher levels of IL-6 leading to increased inflammation and lung injury. Thus, IL-6 may be both a diagnostic marker of AKI and prolonged mechanical ventilation, as well as a potential therapeutic target.

Our study has several strengths. As stated previously, our study subjects were children undergoing CPB surgery. Therefore, the timing of the increase in IL-6 and IL-8 levels relative to the ischemic insult is clear and, as in our animal models, occurs early after injury. Furthermore, this is a well-characterized cohort of children, in whom other plasma and urine biomarkers of AKI have been shown to have excellent predictive value. Our study also has some limitations. Because this is a clinical study, our results are associations and cannot prove causality. However, our results are similar to our prior observations in animal models [9] and suggest that AKI may affect other end organs in human disease through its effects on systemic cytokines. The study population is relatively small and made up of children undergoing CPB, so the generalizability of these results to other populations is unclear. However, given their lack of other comorbidities, this pediatric population has been invaluable for studies of ischemic AKI unconfounded by other diseases that could contribute to a proinflammatory state (*e.g*., sepsis). Further studies in critically ill adult populations are warranted to confirm and extend our findings; however, these studies are likely to be confounded by the contribution of other disease states to systemic cytokine levels.

## Conclusions

We have shown that serum IL-6 and IL-8 levels increase early after CPB and are predictive of AKI in a pediatric population. Based on data from animal models in which AKI itself leads to elevated IL-6 and IL-8 levels, we hypothesize that the increase in IL-6 and IL-8 is because of increased cytokine generation or reduced cytokine clearance in the setting of AKI. Furthermore, among patients with AKI, IL-6 levels are predictive of prolonged mechanical ventilation. This result is similar to our prior results in animal models, in which AKI resulted in higher serum IL-6 levels and concomitant lung injury. Thus, serum cytokines may have an important role as early biomarkers for AKI, as well as a potential role as a therapeutic target in AKI. Modulation of these cytokines may reduce the degree of kidney injury itself, as well as the deleterious effects of kidney injury on other end organs, including the lung.

## Key messages

• The proinflammatory cytokines IL-6 and IL-8 are increased early (at 2 hours) in patients with AKI due to CPB.

• Other serum cytokines, including IL-18, are not increased in patients with AKI.

• Among patients with AKI, serum IL-6 predicts prolonged mechanical ventilation.

• Serum IL-6 and IL-8 may be useful early biomarkers to detect AKI and predict complications (*i.e*., prolonged mechanical ventilation).

## Abbreviations

AKI: acute kidney injury; CPB: cardiopulmonary bypass; ELISA: enzyme-linked immunoabsorbent assay; G-CSF: granulocyte colony-stimulating factor; GM-CSF: granulocyte-macrophage colony-stimulating factor; IFN: interferon; IL: interleukin; ROC: receiver operator curve; TNF-α: tumor necrosis factor-α.

## Competing interests

KDL, CA, GS, CDK and SF have no competing interests to disclose. CE holds US Patent 7,141,382 for IL-18 as an early biomarker of AKI. PD is on the Advisory Board of Abbott Diagnostics and Biosite, Inc., and has licensing agreements with Abbott and Biosite for developing NGAL as a biomarker for acute renal failure.

## Authors' contributions

KDL performed the statistical analysis and drafted the manuscript. CA carried out biomarker measurements. GS performed the initial statistical analysis. CE was responsible for the serum IL-18 analyses and participated in the design of the study. CDK was responsible for recruiting the patients, obtaining the samples, and maintaining the clinical database. PD designed and carried out the original cohort study of children undergoing CPB and participated in the design of this study. SF conceived of the study, participated in its design and coordination, performed biomarker measurements, and drafted the manuscript. All authors read and approved the final manuscript.

## Supplementary Material

Additional file 1 The following additional data are available with the online version of this article. Additional data file 1 is a table listing the cardiac surgical procedures performed in children in this cohort. Click here for file
