# Supplementary material for: Serum Interleukin-6 and interleukin-8 are early biomarkers of acute kidney injury and predict prolonged mechanical ventilation in children undergoing cardiac surgery: a case-control study
Source: Crit Care. 2009 Jul 1;13(4):R104. doi: 10.1186/cc7940 (PMC2750143; doi:10.1186/cc7940)
Supplement: Additional file 1 — The following additional data are available with the online version of this article. Additional data file 1 is a table listing the cardiac surgical procedures performed in children in this cohort. [file cc7940-S1.doc]

**Supplemental Table 1. Cardiac surgical procedures in children with and without acute kidney injury. Procedures differed between the two groups (Fisher exact p <0.001), perhaps due to differences in cardiopulmonary bypass time between the groups (mean CPB time 87 ± 44 minutes in those without AKI compared to 136 ± 63 minutes in those with AKI, p =0.007).**

| Surgical procedure | No acute kidney injury  (N=21) | Acute kidney injury  (N=18) |
| --- | --- | --- |
|  |  |  |
| ASD and VSD repair | 2 | 0 |
| ASD repair | 8 | 0 |
| AV canal repair | 0 | 4 |
| Arterial switch operation | 0 | 2 |
| Bidirectional Glenn procedure | 0 | 1 |
| Coarctation repair | 1 | 0 |
| Fontan procedure | 1 | 3 |
| Mitral valve repair | 1 | 0 |
| Norwood procedure | 0 | 1 |
| Patch aortoplasty | 0 | 1 |
| Pulmonary artery reconstruction | 1 | 0 |
| Tetralogy of Fallot repair | 0 | 1 |
| Total anomalous pulmonary return repair | 0 | 2 |
| Transitional AV canal | 1 | 0 |
| Truncus arteriosus repair | 2 | 0 |
| VSD and aortopulmonary window repair | 0 | 1 |
| VSD and pulmonic stenosis repair | 1 | 0 |
| VSD repair | 3 | 2 |
